# Gland New Psychosis: New Onset Adult Psychosis with Suicidal Ideation and Attempt in the Setting of Thyroid Storm

**DOI:** 10.1155/2017/7402923

**Published:** 2017-03-07

**Authors:** Esteban Cota, Jacob Lentz

**Affiliations:** ^1^David Geffen School of Medicine at UCLA, 10833 Le Conte Ave, Los Angeles, CA 90095, USA; ^2^UCLA Medical Center, 757 Westwood Blvd, Los Angeles, CA 90095, USA

## Abstract

We present a case of new onset psychosis in the setting of thyroid storm in a woman with no previous psychiatric history. The patient presented with ongoing suicidal ideation, a suicide attempt that was interrupted by her husband, and audio and visual hallucinations. The patient was placed on a psychiatric hold and treated for thyrotoxicosis as well as psychosis. Treatment of the thyroid hormone overload resulted in a rapid resolution of her symptoms; she was discharged in excellent condition, and she has had no repeat hallucinations or self-injury ideation or attempts since. Although rare, thyrotoxicosis is a potentially life-threatening cause of psychiatric illness and should always be kept on the differential diagnosis for a patient with a first episode of psychosis. This case highlights how thyroid storm physiology, beyond its well-studied hemodynamic and metabolic instability, can be potentially fatal due to psychiatric sequelae. It also highlights the crucial role of a thorough history and physical exam in all patients.

## 1. Introduction

Thyroid storm is a life-threatening emergency that must be recognized and treated promptly to avoid the cardiovascular collapse of its hypermetabolic state. It is characterized by tachycardia and arrhythmias, fevers, gastrointestinal distress, and neurologic and psychiatric agitation. Depending on the presentation, it can mimic intoxication, medication overdose, or a first psychotic break, and thus the possibility of a thyroid etiology must always remain on the differential diagnosis for all clinicians approaching a patient with altered mental status.

## 2. Case Presentation

A 58-year-old female with a history of lupus and plaque psoriasis presented to the emergency department with new onset hallucinations and suicidal ideation, as well as one witnessed attempt to throw herself into traffic. The patient was brought to the hospital after what her husband said were three days of the patient talking to people who were not there, crying frequently, not sleeping, openly expressing a desire to die, and one attempt to run from their home to a nearby highway in order to jump in front of a car. The patient's husband had chased after and restrained her, after which he brought her to the hospital.

The husband relayed that the patient had never before in the course of their 35-year marriage behaved in this manner, and he further insisted that the patient was previously generally “happy and smiling.” In-house hospital records showed a long history of lupus and psoriasis management as well as a recent admission two weeks prior for mastoiditis; she had received IV clindamycin and ceftriaxone and then been discharged on oral clindamycin. There was no record of any mental health history.

The medical records showed that, during the recent hospital admission, the patient was discovered to have a depressed thyroid stimulating hormone (TSH) level; she was diagnosed with hyperthyroidism and treated with methimazole (MMU) and propranolol for presumed Grave's disease. At the time of discharge, she was continued on a half dose of methimazole. Propranolol was discontinued before discharge based on clinical response to treatment, with endocrinology clinic follow-up scheduled for three weeks from the time of discharge.

The patient's husband further relayed that the patient had been searching their home for a knife for the past two days while telling him that she “needed to die,” compelling him to remove all kitchen knives from their home. On interview, the patient was asked why she was looking for a knife and she responded “because I want to die.”

The patient and her husband both denied any recent head trauma. They both further denied any alcohol use or illicit drug use. The patient denied any abdominal pain, diarrhea, dysuria, vomiting, or shortness of breath. She endorsed restlessness, palpitations, and a sensation of “feeling too hot.”

On exam, blood pressure was 145/88, temperature was 37.5, respirations were 17, oxygen saturation was 99 percent on room air, and heart rate was 129 beats per minute. On the cardiac monitor, she was in gross atrial fibrillation, which had previously not been documented in her medical record. During the exam, the patient was noted to have rapid, pressured speech. Voice volume was normal, and her mood was depressed. She became tearful during the interview, and she intermittently appeared to be responding to internal stimuli. Of note, she seemed to be quite open about all her symptoms and was an excellent historian. She freely admitted to suicidal ideation (SI) and audiovisual hallucinations (AVH). She admitted to attempting to run into traffic in order to kill herself. She described hearing what she described as supernatural voices speaking to her; she said that she could see “spirits and lions” that were likewise speaking to her. She did not believe that the voices were telling her to kill herself but rather said that she wanted to die “because I am so sad.” She was oriented to her name only. She did not know the year and believed she was currently in a prison. She asked the doctor for a knife several times. She also said that she felt as though she wanted to start running “because I can't keep my legs from moving.” She had pulled all the blankets off the bed, had removed much of her hospital gown, and asked if she could have some ice water “because it is so hot in here,” despite the fact that the emergency department was air-conditioned. The remainder of the exam was notable for an irregularly irregular heartbeat, clear lungs, tongue fasciculations, tremulousness, diffuse hyperreflexia, and a large, nontender thyroid gland.

The patient was placed on a medical detainment and assigned a one-to-one sitter to monitor her. Psychiatry was consulted. An electrocardiogram was ordered, which showed sinus tachycardia with resolution of the previously noted atrial fibrillation. Urine toxicology was negative. Labs showed a slightly low potassium level, in keeping with a hyperadrenergic state. TSH was undetectable.

It was well past midnight, so the decision was made to treat empirically for thyroid storm. Propranolol 80 milligrams PO and methimazole (MMU) 20 milligrams PO were ordered. The patient took the medication without any resistance. She was given a large cup of ice water, as well as an IV bolus of a liter of normal saline. Endocrinology was consulted, and the head of endocrinology began driving to the hospital from his home to see the patient. Dexamethasone 10 milligrams IV was then given. Meanwhile, the psychiatry service interviewed the patient. They agreed with the plan to treat for hyperthyroidism and placed the patient on a 72-hour psychiatric hold for danger to self and grave disability.

Two hours after treatment was initiated, the patient's heart rate had slowed to 89 beats per minute. The cardiac monitor showed normal sinus rhythm. Tongue fasciculations had stopped, though hyperreflexia remained. The patient said that she felt “so much better” and was at this point denying SI and AVH. She showed insight into her condition, saying “I was feeling very upset and scared. I was seeing lions, and they were talking to me. I was so scared. I could hear voices that I knew weren't there, but I felt so sad about how I felt that I wanted to die.” She wanted to know what medicine she had been given that had made her feel better, and she asked if she could have a prescription for it.

Endocrinology consult arrived and assessed the patient, likewise finding hyperreflexia and an enlarged thyroid gland. Endocrinology made a diagnosis of thyroid storm in the setting of Grave's disease. Both consult services recommended a brain MRI to rule out an intracranial process. This showed no evidence of bleeding or stroke, nor any evidence of lupus cerebritis (which had been included on the differential due to the patient's lupus history).

The patient was admitted to medicine and was followed by psychiatry. The day after admission, she again began endorsing SI and AVH. Free T4 and T3 levels at this time were 2.10 ng/dL and 2.09 ng/dL, respectively. Psychiatry recommended olanzapine (Zyprexa) 5 milligrams daily, which was initiated to good effect. Propranolol, MMU, and steroids were continued and adjusted based on endocrine consult recommendations. Thyroid stimulating immunoglobulin (TSI), ordered as a send-out lab during the patient's previous admission for mastoiditis, returned positive, and the diagnosis of Grave's disease was made.

As the patient's thyroid labs normalized, her psychiatric symptoms resolved. Olanzapine was discontinued at the recommendation of psychiatry. Endocrinology recommended radioactive iodine ablation of her thyroid gland, which was scheduled and then aborted when she was found to have sudden onset pancytopenia, a well-documented side effect of methimazole [1]. Methimazole was discontinued, and her cell lines began to recover. After thorough reassessment, psychiatry lifted the hold, and the patient was discharged from the hospital on a steroid taper and propranolol.

## 3. Discussion

Thyroid hormone is a singularly important hormone in the body. Its deficiency or overabundance can have systemic effects throughout nearly every organ system. The ability of thyroid hormone to affect a patient's psychiatric and neurologic status has been amply characterized in the literature [2]. Indeed, patients with lower free T4 have shown to have poorer prognosis in terms of attention performance after a first episode of psychosis (FEP) [3].

Hyperthyroidism exists clinically on a spectrum from asymptomatic hyperthyroidism that eludes detection to minor symptomatology of heat intolerance, palpitations, and GI upset to a life-threatening thyroid storm. Thyrotoxicosis is the clinical syndrome of organ systems exposed to high levels of circulating thyroid hormone [4]. Thyroid storm is defined as an “acute, exaggerated manifestation of a thyrotoxic state in patient” [2]. It is a life-threatening emergency requiring immediate intervention to prevent circulatory and metabolic collapse [5]. It represents the extreme spectrum of a thyrotoxic state wherein collapse of organ function may suddenly occur [6]. Most commonly, the underlying etiology of thyroid storm is Grave's disease, though the progression from thyrotoxicosis to frank thyroid storm is often precipitated by a new insult [2]. Of note, in the present case the patient was treated for mastoiditis shortly before the onset of her symptoms.

A thyroid storm is a clinical diagnosis. The Burch-Wartofsky Score, created by two expert endocrinologists in 1993, can be used as a clinical decision-making tool for diagnosing thyroid storm (see Table 1). Based on the patient's history and initial exam, her score was 60, which is highly suggestive of a thyroid storm. Of course, this score did not exclude other etiologies; the key to the Burch-Wartofsky Score is being aware that it exists and, in the right clinical context, warrants consideration.

There are several treatment options for thyroid storm [4]. Propranolol, in addition to blunting the adrenergic effects of thyroid hormone, also blocks the peripheral conversion of T4 to T3, the metabolically active form of thyroid hormone [5]. Methimazole and propylthiouracil (PTU) both inhibit thyroperoxidase in the thyroid gland, essentially shutting down the production of thyroid hormone [6]. PTU also, unlike methimazole and like propranolol, blocks the peripheral conversion of T4 to T3. Both methimazole and propylthiouracil are known to precipitate agranulocytosis [1]. Corticosteroids likewise block the peripheral conversion of T4 to T3, and their long-acting effect make them a first-line agent in thyroid storm management [6].

Another treatment option of thyroid storm, counterintuitively, is to give the patient iodine (the key element in the production of thyroid hormone) [2]. Through the Wolff-Chaikoff effect, high dose iodine shuts down the thyroid gland's production of thyroid hormone. Treatment options include sodium iodine 0.5 milligrams IV BID and Lugol's Solution 8 drops PO QID [2].

Fevers in a thyroid storm can be managed symptomatically with antipyretics, and psychosis can be controlled in the acute setting with antipsychotic medication; with both, however, the key is the management of thyroid hormone burden [5]. It is also important to manage the patient's fluid status, as the hypermetabolic state of thyrotoxicosis/thyroid storm results in increased insensible fluid losses [6].

Another consideration in the setting of altered mental status in the setting of thyroid disease is Hashimoto's encephalopathy. This rare entity is a diagnosis of exclusion and of uncertain etiology. It presents with varied neuropsychiatric manifestations that can include psychosis, delirium, and seizure activity [7]. Antithyroid antibodies and/or thyroid peroxidase antibodies are invariably present in this condition, and it is managed primarily with thyroid hormone replacement and steroids, though antipsychotics and antiepileptics can also be used. In a case of Hashimoto's encephalopathy, both MRI and CSF studies would be normal. Differentiating it from thyroid storm would depend on thyroid levels and the absence of other clinical signs of thyrotoxicosis [7].

Another specific aspect of this case warrants further discussion. The patient had two previously diagnosed autoimmune diseases, systemic lupus erythematosus (SLE), and plaque psoriasis; her newfound Grave's disease added a third. The first two were previously confirmed on serology and managed at the hospital in question, and the latter was confirmed via the presence of thyroid stimulating immunoglobulin (TSI). The frequency of concomitant autoimmune disorders and a sex-linked predominance has been well-studied and characterized [8–10]. What is intriguing about this case is that the patient's autoimmune crisis, a thyroid storm, came in the setting of a recent receipt of antibiotics.

While correlation certainly does not prove causation, the temporal relationship is intriguing, and it may warrant further study. The immunoactive role of the gut has long been acknowledged, and, in recent years, the effects of gut flora on the body's immune system have become increasingly clear if still poorly understood [10–12]. In this case, a patient received antibiotics for mastoiditis, which may well have altered the balance of her gut flora (IV antibiotics can affect the gut via hepatic clearance). It is the alteration in the composition of gut flora, that is, for instance, the precipitant of* Clostridium difficile* infection (interestingly, the patient received clindamycin, the most notorious culprit in* C. difficile* infections). Shortly thereafter, she entered an autoimmune crisis with near-fatal psychiatric manifestations.

Thyrotoxicosis aside, there is a well-documented correlation between autoimmune disease and schizophrenia [13, 14]. That a nexus with gut flora exists has also been proposed [14]. In the present case, we see a woman with known autoimmune disease receiving an antibiotic that is famously toxic to normal gut bacteria, and shortly thereafter a thyroid storm begins. Whether it is due to undertreatment of her hyperthyroidism or perhaps due to a rapid alteration of her gut flora is beyond the scope of this paper. But the connection is intriguing nevertheless. Is it possible that, in the future, probiotics that may help restore normal gut flora could become routine adjuncts for autoimmune patients receiving antibiotics? Only time will tell.

## 4. Conclusion

The patient had no further episodes of hallucinations. She was followed by both the endocrinology and psychiatry services as an outpatient. Follow-up blood work showed a resolution of her pancytopenia, and she underwent ablation of her thyroid gland and was started on levothyroxine.

The patient continues to do well and has had no further psychiatric issues.

## Figures and Tables

**Table 1:** Burch-Wartofsky thyroid storm scoring system.

	Points
Temperature	
37.2–37.7	5
37.8–38.2	10
38.3–38.8	15
38.9–39.4	20
39.4–39.9	25
>40	30
CNS effects	
Mild (agitation)	10
Moderate (delirium/psychosis)	20
Severe (seizure/coma)	30
GI symptoms	
Mild (diarrhea)	10
Severe (jaundice)	20
Tachycardia	
99–109	5
110–119	10
120–129	15
130–139	20
>140	25
CHF symptoms	
Mild	5
Moderate	10
Severe	15
Atrial fibrillation	
Absent	0
Present	10
Precipitant history	
Negative	0
Present	10

Total score	
<25: thyroid storm unlikely	
25–44: suggestive of thyroid storm	
>45: highly suggestive of thyroid storm	
